# Effects of feeding pomegranate seed pulp and coconut meal by-products on milk yield, milk quality, and metabolic responses of Awassi ewes and pre-weaning growth

**DOI:** 10.14202/vetworld.2024.1149-1156

**Published:** 2024-05-17

**Authors:** Belal S. Obeidat, Manal H. Qadorah, Milton G. Thomas

**Affiliations:** 1 Department of Animal Production, Faculty of Agriculture, Jordan University of Science and Technology, P.O. Box 3030, Irbid, 22110, Jordan; 2 Texas A&M AgriLife Research, Beeville, Texas, 78102, USA

**Keywords:** Awassi ewes, coconut meal, milk yield and quality, pomegranate seed pulp

## Abstract

**Background and Aim:**

Feeding by-products, such as pomegranate seed pulp (PSP) and coconut meal (COC), to livestock may enhance production efficiency and increase profits. This study aimed to evaluate the effects of PSP and COC on milk production, body weight change, metabolic response (Exp. 1), digestibility, and N balance (Exp. 2).

**Materials and Methods:**

Twenty-four ewes nursing single lambs were randomly assigned to one of three diets: Control (CON) (n = 8), 7.5% PSP (n = 8), and 7.5% COC (n = 8) of dry matter (DM). Every sheep was born 3–4 days before the start of the experiment. The 1^st^ week of the experiment was devoted to diet adaptation, while the data were collected during the following 8 weeks (Exp. 1). Nine lambs were randomly assigned to one of the three diets for intensive data collection to evaluate the diet digestibility and animal nitrogen (N) balance (Exp. 2). The data were analyzed using the MIXED SAS procedures.

**Results:**

The PSP group consumed more DM, followed by the COC and CON groups. For lambs, the final BW, total gain, and average daily gain in the PSP group were greater (p < 0.05) than those in the COC and CON groups. The CON group had lower milk output, total solids, protein, fat, and lactose levels than the PSP and COC groups (p < 0.05). The cost of milk production was lower (p < 0.05) in the PSP and COC diet groups than in the CON group. Blood serum parameters were similar among the dietary groups, except for total cholesterol and high-density lipoprotein, which were higher in the COC group than in the CON group. No differences were observed in nutrient digestibility and N balance.

**Conclusion:**

Feeding PSP and COC to nursing ewes appears beneficial because it increases milk production and pre-weaning lamb growth and reduces milk production cost.

## Introduction

Sheep (*Ovis aries*) play a significant social and economic role in maintaining food security worldwide. This is the case in Jordan, where sheep farmers are affected by high prices and the lack of availability of feed due to the need to import most feed [1]. In recent years, feed prices have increased to such an extent that sheep production is potentially economically unfeasible due to the lack of feed production in Jordan and the high cost of transport for imported feed [2]. As conventional imported feed ingredients may negate profit margins for sheep, livestock producers have begun to search for alternative feeds at lower costs [3]. This research program conducted a series of experiments and found that the use of alternative feeds decreases the cost of production without impacting the health status of small ruminants [2–6]. Feeding by-products from local sources, such as pomegranate seed pulp (PSP) and coconut meal (COC), to livestock can reduce the cost of milk production and increase profits. In addition to the availability of PSP and COC, by-products from plant oils and juices provide livestock producers with the possibility of substituting these by-products for conventional diet ingredients.

Pomegranate (*Punica granatum* L.) is a tropical and subtropical deciduous shrub indigenous to the Iranian Plateau, the Himalayas in Northern Pakistan, and Northern India. They are divided into three parts: Seeds, juice, and peels, which contain the husk and internal membranes [7]. Pomegranate has beneficial nutritional properties for humans because it contains secondary metabolites and natural products which may be beneficial for humans and animals. Pomegranate components have been shown to have antimitotic, antioxidant, antibacterial, anti-inflammatory, and immunomodulatory benefits [8]. In addition, phenolic chemicals such as hydrolyzable tannin, anthocyanin, and flavonoid glycosides found in pomegranate byproducts are capable of significant radical scavenging [8]. Pomegranate seeds, which form more than 60% of the pomegranate’s weight, are used as a source of oil and/or juice, with the by-products known as PSP [9]. The use of pomegranate by-products in livestock feed can reduce the negative effects of waste disposal in the production of pomegranate juice and oil. Therefore, it can also be used as a low-cost alternative ingredient for costly grains in sheep feeding. For example, Hamed and Abdel-Tawwab [10] found that feeding dried pomegranate peel at 1% improved growth performance, reduced diet costs, and enhanced fish economic efficiency. The nutritional significance of pomegranates and their by-products in ruminant nutrition has been the subject of numerous studies [3, 11]. PSP has no influence on growth performance, carcass characteristics, and meat quality [11].

Coconut (*Cocos nucifera*) is a tropical fruit that was first cultivated in South-east Asia. However, COC is considered to be a substantial protein supplement in livestock feed [12]. COC supplementation has a superior effect on the dressing percentage and higher slaughter weight in growing lambs [13]. In addition, coconut is a tropical fruit that is grown in more than 80 countries worldwide. The world’s annual coconut production has been reported to be 63.68 million tons, resulting in approximately 2.54 million tons of COC [14]. Coconut is used as a source of oil, which converts more than 70% of the weight of coconut into by-products [15]. COC by-product (i.e., COC) is considered as a usable protein supplement in livestock feedstuffs because it contains crude protein (CP) (22.75%), essential amino acids, including lysine (0.59%) and methionine (0.34%), crude fiber (CF) (12.11%), ether extract (EE) (2.89%), and 7.41% of total ash with dry matter (DM) [15]. In a recent study, Rokhayati [16] reported that feeding grazing goats COC at 50% of the supplement did not affect their body weight (BW) change or DM intake. Even though the protein content in COC is low compared to the commonly used sources of protein, it is economically important in tropical regions where the feed supply (particularly protein supplement feed) is a constraint in livestock production; COC has 50.7% apparent digestibility of protein [17] when used in swine diets.

In this study, we hypothesized that ewes receiving PSP or COC in their diets will have a comparable performance compared to ewes receiving the control (CON) diet. To test this hypothesis, Awassi ewes and their lambs were evaluated for the effects of PSP and COC in their diets on milk production, BW change, metabolic response (Exp. 1), digestibility, and N balance (Exp. 2).

## Materials and Methods

### Ethical approval

After receiving approval from the JUST Institutional Animal Care and Use Committee (#: 459/12/04/16A), two experiments were conducted at JUST facilities. In the case of mature ewes, a farm veterinarian examined each experimental animal before the start of the trial to assess its health and udder condition. The animals were routinely examined throughout the studies.

### Study period and location

The study was conducted from October 2022 to December 2022 at the JUST Agricultural Research and Training Unit/Faculty of Agriculture.

### Animals

Lactating Awassi ewes and their nursing lambs were used to determine the effects of PSP or COC on milk production, milk composition, feed consumption, BW (Exp. 1), digestibility, and nitrogen (N) balance (Exp. 2).

### Experiment 1

To comply with the National Research Council [18] guidelines for lactating ewes, 24 newly lambed Awassi ewes (initial average BW = 51.6 ± 2.67 kg; age = 5–6 years; 3–4 parities) and their single lambs were randomly assigned to one of three treatments: CON (CON: n = 8), 7.5% PSP (PSP: n = 8), and 7.5% COC (COC: n = 8) of DM. Every sheep gave birth 3–4 days before the start of the experiment. PSP and COC were used to replace part of barley grain and soybean meal. The chemical compositions of PSP and COC were 94.0% and 93.2% DM, 17.8% and 19.8% CP, and 34.3% and 67.2% neutral detergent fiber (NDF), 20.3% and 44.4% acid detergent fiber (ADF), and 11.3% and 7.64% EE. The diet was designed to provide 16% CP and to be isonitrogenous. Table-1 summarizes the components and chemical compositions of the experimental diets. Individual pens (1.5 m × 0.75 m) were used to confine ewes and their lambs. The pens were equipped with plastic feeders and water troughs that provided unlimited access to food and water. The study lasted for 9 weeks. The 1^st^ week was used to adapt the animals to the diets and pens, and the remaining 8 weeks were used to collect data. The ewes received their experimental diets daily at 9:30 am and had unlimited access to water throughout the study period. The ewes and lambs were weighed separately every 2 weeks. To prevent feed waste, feed buckets were regulated in such a way as to deliver 110% of the daily feed intake. Feed refusals were weighed, documented, and sampled daily to precisely monitor the nutritional intake.

**Table-1:** Ingredients and chemical composition of diets that included PSP or COC fed to Awassi sheep (Exp. 1 and Exp. 2).

Item	Diet^1^		

	CON	PSP	COC
Ingredients (% of DM)			
Barley grain, whole	46.0	40.0	40.5
Soybean meal, 440 g/kg CP (solvent)	22.0	20.5	20.0
Wheat straw	30.0	30.0	30.0
Pomegranate seed pulp	0	7.5	0
Coconut flower	0	0	7.5
Salt	1.0	1.0	1.0
Limestone	0.9	0.9	0.9
Vitamin-mineral premi×^2^	0.1	0.1	0.1
Cost (US$/1,000 kg) ^3^	452	421	419
Nutrients (% of DM)			
DM	90.5	91.0	91.3
CP	15.9	15.8	15.7
NDF	33.2	36.8	34.4
ADF	14.9	17.8	16.0
EE	0.9	1.8	1.7

1 Diets: (1) Control (CON); (2) 7.5% PSP (PSP); and (3) 7.5% COC (COC) of dietary dry matter.

2 Composition per kg contained: Vitamin A=600,000 IU, Vitamin D3=200,000 IU, Vitamin E=75 mg, Vitamin K3=200 mg, Vitamin B1=100 mg, Vitamin B5=500 mg, Lysine=0.5%, DL-methionine=0.15%, Manganese oxide=4000 mg, Ferrous sulphate=15,000 mg, Zinc oxide=7000, Magnesium oxide=4000 mg, Potassium iodide=80 mg, Sodium selenite=150 mg, Copper sulphate=100 mg, Cobalt phosphate=50 mg, Dicalcium phosphate=10,000 mg.

3 Calculated based on ingredient prices during 2023. CON=Control, PSP=Pomegranate seed pulp, COC=Coconut meal, DM=Dry matter, CP=Crude protein, NDF=Neutral detergent fiber, ADF=Acid detergent fiber, EE=Ether extract

Milk release was induced by intravenously administering 0.75 mL oxytocin to evaluate milk yield, and ewes were then milked by hand at 08:00 by experienced laborers. A period of 3 h was left before the ewes were subjected to the second dose of oxytocin and milked again, and the milk yield was documented. During the two dosage periods, lambs were kept away from their mothers [19]. The milk yield was calculated over a period of 24 h (3 h × 8). The milk samples (125 mL/ewe) were tested for total solids, CP (Kjeldahl technique; N × 6.38), and fat (Gerber method) using a forced oven set to 55°C. The cost of milk production ($US/kg of milk) was calculated by multiplying the feed efficiency (DM intake daily milk yield) with the price per kg of diet.

Diet samples and refusal samples were stored in the freezer at –20°C during the study. After completion of the study, the samples were thawed and composited to obtain one sample for each diet and one refusal sample for each animal. The samples were then dried in an oven at 105°C to evaluate the DM content [20]. CP was measured using the Kjeldahl method (CP: N × 6.25). NDF and ADF were examined using an ANKOM^2000^ fiber analyzer (ANKOM Technology Corp., Fairport, NY, USA). The EE content was evaluated using the Soxtec procedure (SXTEC SYSTEM HT 1043 Extraction Unit, TECATOR, Box 70, Hoganas, Sweden).

Blood samples were obtained from the jugular vein at the beginning and end of the experiment (before feeding) at 8:00 a.m. using plain vacutainers. Blood samples were centrifuged at 1008× g for 15 min at room temperature (24°C) for 1 h. Serum samples were rapidly separated and stored at –20°C until the day of analysis. A spectrophotometer (JENWAY 6105 UV/Vis, Model 6105, Janeway LTD Felsted, Dunmow ESSEX CM6 3LB, UK) using commercial kits (Biolabo S.A.S., Less Hautes Rivers, Maizy, France) was used to measure the concentrations of various substances in the serum, including glucose, urea nitrogen, total cholesterol, high-density lipoprotein (HDL), low-density lipoprotein, triglycerides, alanine aminotransferase, and aspartate aminotransferase.

Statistical Analysis System (SAS) (version 8.1, 2000, SAS Inst. Inc., Cary, NC, USA) was used to analyze the data using MIXED procedure. The milk production and the ewe and lamb BW were examined using analysis of variance for repeated measures in a model that included treatment, week, and the treatment week. Only the primary effects are discussed because no interaction between treatment and week was observed. Treatment had the only fixed effect on diets, milk composition and yield, nutritional intake, and ewe and lamb BW. A statistically significant difference was defined as one with a probability of p ≤ 0.05. The following model was used for the analysis.

Y_ijk_ = μ + B_i_ + ε_ij_

Specifically, Y_ijk_ is the dependent variable, μ is the overall mean, B_i_ is the treatment effect, and ε_ij_ is the random error.

### Experiment 2

Nine Awassi lambs (BW = 27.4 ± 1.65 kg; age = 4–5 months) were randomly assigned to one of the three diets used in Exp. 1 (3 lambs/diet) to examine the nutritional digestibility and N balance of soybean meal and barely grain partially substituted with PSP or COC. The Awassi lambs were weighed and their ears were marked before the trial started. The study was conducted for 21 days in individual shaded pens (0.75 m × 1.5 m), with the adaptation phase lasting 14 days, while the data collection phase lasted 7 days and was conducted in metabolic cages (0.8 m × 1.05 m). In the shaded cages, unlimited access to food and water was made possible by plastic feed and water troughs.

To prevent feed waste, feed buckets provided 110% of the daily voluntary feed consumption. Feed refusals were weighed, recorded, and sampled to accurately assess the nutritional intake. Weekly refusals were sampled, collected, and evaluated for DM, CP, NDF, and ADF. From days 15–21, the lambs were housed in metabolic cages with fecal trays allowing complete fecal and urine collection. Daily fecal output was collected, weighed, and recorded, and 10% was kept for subsequent analysis. Urine samples were collected, weighed, and recorded using plastic containers, and 5% was kept to evaluate the N retention. Each bottle contained 50 mL of 6N HCl to prevent ammonia loss. All samples were dried at 55°C in a forced-air oven to reach a constant weight, air equilibrated, and ground in a Wiley mill (Brabender OHG Duisdurg, Kulturstrase 51-55, type 880845, Nr 958084, Germany) to pass through a 1-mm screen and were stored for further analysis. Dietary and fecal samples were tested for DM, CP, NDF, and ADF as specified in Experiment 1.

Urine samples collected from each lamb were mixed, and the N content was calculated using the Kjeldahl method. N intake was calculated by dividing the DM consumption by the amount of N in each diet. We calculated the amount of N lost in feces and urine by multiplying the N content in the feces and urine by the fecal and urinary outputs, respectively. The retained N (g/day) was then calculated by deducting the daily N intake from the fecal and urine production. N retention (%) was obtained by dividing the retained N by the N intake.

The SAS MIXED technique (version 8.1, 2000, SAS Institute Inc., Cary, NC, USA) was used to analyze the data, with the lamb as the random variable. The dietary treatment was the fixed effect in these analyses. MIXED SAS algorithms were applied for the least square means to separate the means. An alpha value of p = 0.05 was used as an indicator of differences in experiments 1 and 2.

## Results

### Experiment 1

Table-2 presents the DM intake and BWs of the ewes and lambs. The intake of DM was the highest (p < 0.05) in ewes fed the PSP diet, followed by those fed the COC diet, and the lowest in ewes fed the CON diet. Changes in initial BW, final BW, and BW of the ewes were similar between the dietary groups (p > 0.05). However, the final BW, total gain, and ADG were the highest in the PSP group (p < 0.05), followed by the COC and CON groups.

**Table 2:** Effects of feeding PSP or COC on the dry matter intake and BW changes of the Awassi ewes and the growth of their lambs (Experiment 1).

Item	Diet^1^			

	CON (n = 8)	PSP (n = 8)	COC (n = 8)	SEM^2^
DM intake, g/d	2157^a^	2282^c^	2232^b^	14.8
Ewes				
Initial BW (kg)	50.8	52.6	51.4	2.67
Final BW (kg)	50.0	51.8	51.8	2.27
BW change (kg)	−0.75	−0.88	0.38	1.125
Lambs				
Initial BW (kg)	6.1	6.1	5.9	0.53
Final BW (kg)	15.8^a^	20.5^c^	18.3^b^	0.87
Total gain (kg)	9.8^a^	14.4^c^	12.4^b^	0.85
Average daily gain (g)	175^a^	257^c^	222^b^	0.53

1 Diets: (1) Control (CON); (2) 7.5% PSP (PSP); and (3) 7.5% (COC) of dietary dry matter.

2 SEM=Standard error mean.

^a,b,c^Means within a row without a common superscript differ significantly (p < 0.05). CON=Control, PSP=Pomegranate seed pulp, COC=Coconut meal, DM=Dry matter, BW=Body weight

Table-3 shows milk production, milk composition, milk composition yield, and blood metabolite content. The total milk solid content was the highest (p < 0.05) in the COC group, followed by the PSP group and then the CON group. Milk protein and lactose contents were similar (p > 0.05) in all treatments. The milk fat content was lower (p < 0.05) in the CON group than in the PSP and COC groups. Daily milk yields (i.e., total solids, protein, fat, and lactose) were greater in the PSP and COC groups than in the CON group (p < 0.05). Feed-to-milk yield was reduced (p < 0.048) in the PSP and COC groups compared to the CON group. The milk cost decreased (p 0.05) in the groups consuming PSP and COC compared with the CON group. No differences (p > 0.05) were observed in the blood metabolites when PSP and COC were fed, except for the cholesterol and HDL content: the group consuming COC had greater (p = 0.05) cholesterol and HDL content compared with the CON group, whereas the PSP was intermediate (Table-4).

**Table 3:** Effect of feeding PSP or COC on milk yield and milk composition of Awassi ewes fed lactation diets (Experiment 1).

Item	Diets^1^			

	CON (n = 8)	PSP (n = 8)	COC (n = 8)	SEM^2^
Milk yield, g/d	1435^a^	1735^b^	1771^b^	96.9
Milk composition, %				
Total solids	18.3^a^	19.76^b^	20.5^c^	0.24
Protein	4.5	4.5	4.6	0.04
Fat	7.1^a^	8.5^b^	9.0^b^	0.26
Lactose	6.7	6.0	6.1	0.04
Milk yield, g/d				
Total solids	261.6^a^	342.6^b^	366.1^b^	19.89
Protein	64.0^a^	78.5^b^	81.2^b^	4.60
Fat	101.4^a^	147.6^b^	161.4^b^	9.58
Lactose	87.7^a^	104.2^b^	109.1^b^	6.09
Feed to milk yield	1.51^b^	1.37^a^	1.27^a^	0.048
Milk cost ($US/kg)	0.68^b^	0.58^a^	0.53^a^	2.955

1 Diets: (1) Control (CON); (2) 7.5% PSP (PSP); s and (3) 7.5% COC (COC) of dietary dry matter.

2 SEM=Standard error mean. ^a,b,c^Within a row, means without a common superscript differ (p ≤ 0.05). CON=Control, PSP=Pomegranate seed pulp, COC=Coconut meal

**Table 4:** Effect of feeding PSP or COC on blood metabolites of Awassi ewes fed lactation diets (Experiment 1).

Item^2^	Diet^1^			

	CON (n = 8)	PSP (n = 8)	COC (n = 8)	SEM^2^
Blood urea nitrogen, mg/dL	18.64	17.10	19.89	1.245
Glucose, mg/dL	82.25	77.81	75.63	3.210
Cholesterol, mg/dL	56.13^a^	63.50^ab^	75.06^b^	3.931
HDL, mg/dL^b^	42.13^a^	45.44^ab^	55.69^b^	3.095
LDL, mg/dL	11.10	13.85	14.56	2.930
Triglycerides, mg/dL	14.50	16.06	16.56	2.703
Creatinine, mg/dL	0.71	0.72	0.81	0.036
AST, U/L	35.75	42.63	39.13	2.457
ALT, UL	11.81	12.56	13.69	0.925
ALP, UL	55.94	53.56	50.69	4.744

1 Diets: (1) control (CON); (2) 7.5% PSP (PSP); and (3) 7.5% COC (COC) of dietary dry matter.

2 HDL=High-density lipoprotein, LDL=Low-density lipoprotein, AST=Aspartate aminotransferase, ALT=Alanine aminotransferase, ALP=Alkaline phosphatase, SEM=Standard error mean. ^ab^Within a row, means without a common superscript differ (p ≤ 0.05).

### Experiment 2

Table-5 presents the nutrient digestibility and N balance results. The digestibility of DM was greater (p < 0.05) in ewes fed the CON and COC diets. However, no differences (p > 0.05) were observed in CP, NDF, ADF, or EE digestibilities among the dietary groups. N intake was greater (p < 0.05) for the PSP and COC diets than for the CON diet. However, no differences (p > 0.05) were observed in fecal and urinary loss among the diets. Retained N (g/d) and retention N (g/100 g) were greater in the PSP and COC diets than in the CON diet.

**Table 5:** Effect of feeding PSP or COC on the nutrient digestibility and N balance of Awassi ewe lambs (Experiment 2).

Item	Diets^1^			

	CON (n=3)	PSP (n=3)	COC (n=3)	SEM^2^
Digestion coefficient				
DM	78.7^a^	79.7^a^	82.8^b^	0.80
CP	82.2	81.3	84.2	2.06
NDF	62.1	65.6	71.0	2.85
ADF	55.1	60.5	66.8	2.87
EE	84.9	82.7	85.0	2.68
N balance				
N intake, g/d	20.8^a^	23.8^b^	23.7^b^	0.68
N feces, g/d	3.7	4.3	3.8	0.38
N urine, g/d	7.1	5.1	5.5	0.56
N retained, g/d	10.0^a^	14.4^b^	14.5^b^	1.20
N retention, g/100 g	47.8^a^	60.6^b^	61.2^b^	4.02

1 Diets: (1) Control (CON); (2) 7.5% PSP (PSP); and (3) 7.5% COC (COC) of dietary dry matter.

2 SEM=Standard error mean. ^ab^Within a row, means without a common superscript differ (p < 0.05). CON=Control, PSP=Pomegranate seed pulp, COC=Coconut meal, DM=Dry matter, NDF=Neutral detergent fiber, ADF=Acid detergent fiber, EE=Ether extract

## Discussion

The lack of feed and, in particular, the lack of protein is a major factor that affects livestock production. In this study, we attempted to use alternative feeds to replace some of the expensive and less abundant cereal grains found in ruminant rations. In these experiments, nursing ewes and their lambs did not experience any health problems or digestive disorder.

Several important influences on DM intake by ewes were observed in the PSP group compared to the CON group. This observation agrees with El-Elaime [21] and Sadq *et al*. [22], who found that adding 1%–2% of dried pomegranate by-product improved nutrient intake, resulting in better animal performance and feed utilization. The pomegranate’s phenol content also had a positive effect on the population of bacteria in the rumen and the production of microbial protein, resulting in an increase in volatile fatty acids due to its phenol content. Shaani *et al*. [23] reported that high levels of pomegranate peel, fresh, or dry, at up to 8% of the diet may inhibit proteolytic digestive enzymes in the lower gastrointestinal tract. This inhibition may limit amino acid absorption and, consequently, reduce nutrient digestibility, milk yield, and animal performance due to the tannin content, which may have a negative impact on palatability and protein digestibility.

In addition to improved DM intake, an increase in milk fat and total solids was observed in the ewes that were fed the PSP diet. These results disagree with the findings of Modaresi *et al*. [24], who reported that PSP diet had little impact on DM intake, milk yield, and milk solids yield when PSP was fed to lactating goats at 0%, 6%, or 12% of the DM basis. Shabtay *et al*. [25] reported that using 2% and 4% concentrated pomegranate extract increased the milk protein yield compared to the CON diets. In addition, Hassan *et al*. [26] supported our findings as their observations noted an increasing DM, CP, CF, N-free extract, and EE digestibility when 150 g of a pomegranate by-product supplement was included in the feed. Jang *et al*. [27] reported that pomegranate by-product supplements enhanced average daily gain, weight, and growth performance due to the consumption of natural antioxidant(s) that protect the intestinal mucosa against oxidative damage and pathogens. Moreover, the addition of COC was found to improve the ewes’ milk production, whereas coconut consumption did not affect the milk production and composition, as reported by Faciola and Broderick [28]. On the other hand, others reported a reduction in the milk production and composition of ruminants when coconut was added to their diets, which was a result of the decreased intake of DM by those experimental animals [29, 30].

Feeding coconut and pomegranate by-products to ruminants has previously been found to have no important effect on blood metabolites and kidney-functioning enzymes [31–34]; similarly, in the current study, the blood metabolites were not affected by the inclusion of COC and PSP. Nevertheless, the blood cholesterol and HDL content increased in ewes who consumed diets containing COC. Hu *et al*. [31] reported that feeding coconut oil to nursing calves elevated their blood cholesterol levels due to the high concentration of saturated fatty acids in coconut. Moreover, another previous study by Durand *et al*. [35] pointed out that high HDL content was related to differences in fatty acid composition of coconut oil.

Similar to the results of the current study, Zeweil *et al*. [36] reported an increase in cholesterol and HDL content when pomegranate was consumed by rabbits. Obeidat *et al*. [3] reported no effect of PSP on blood cholesterol content, whereas HDL levels increased in sheep. According to Khan *et al*. [37], the inclusion of pomegranate in sheep diets had no impact on the serum HDL content. Pomegranate has also been reported to protect HDL against oxidation [38] and to increase the associated HDL enzyme activity, which breaks down oxidized lipids and hence protects lipoproteins. The contradiction between these studies may be due to differences in the percentage of pomegranate residues contained in the diets, the composition of the diets, or the type and age of the animals. According to these findings, adding COC and PSP to ewes’ diets had no negative effect on their health; consequently, it appears to be safe to include COC and PSP in sheep-formulated diets.

In our study, we observed an improvement in the BW change of the animals fed COC, which supports the results observed by Areghoen [39]. The findings of the current study are also consistent with those of Hennessy *et al*. [40], which involved Hereford steers. In particular, COC enhanced the daily gain, resulting in an increase in the live weight throughout the trial. Rokhayati [16] reported a higher feed conversion ratio and, therefore, high average daily gains in goats fed COC. Consistent with our results, Emami *et al*. [41] and Modaresi *et al*. [24] found no effects of PSP inclusion in the diet on productive traits in kids and goats, which is consistent with our results. The benefits of COC may, therefore, be the most beneficial for sheep.

Kotsampasi *et al*. [42] found that the addition of pomegranate by-product silage had no impact on the DM intake, growth rate, or feed conversion ratio despite the high phenolic content of the mixed rations. PSP could be used to partially substitute cereal grains in growing lambs’ diets at rates of up to 23.5% DM of concentrate, given the lack of adverse effects on the DM intake, growth rate, and lamb performance that has been discovered in numerous trials [10].

In Experiment 2, an important effect was observed regarding DM digestibility and higher N retention in animals fed with COC, which supports the findings of Galgal *et al*. [43], who observed that copra expeller pellets consumed had an increasing N intake. Therefore, N retention due to the high ammonia rumen concentrations led to enhanced efficiency of microbial protein synthesis. DM digestibility may limit peristaltic activity and prevent diarrhea [43]. Few studies have investigated the effects of COC on nursing ewes and the nutrient digestibility and N balance of ewes during the early lactation period, and minimal information has been offered regarding the optimal level at which COC can be incorporated with other ingredients of ruminant rations; therefore, further research is necessary.

## Conclusion

In this study, we tested the hypothesis that feeding Awassi ewes PSP and COC by-products will have a positive impact on various performance measures. The two experiments were found to support this hypothesis; therefore, the results show that adding PSP and COC by-products to rations positively affected the ewes’ feed intake, milk yield, and milk composition, leading to an increase in the BW of their lambs. The replacement of conventional feed with alternative agro-industrial by-products can therefore be considered to be a successful way of reducing feeding costs while addressing the requirement to recycle waste.

## Authors’ Contributions

BSO, MHQ, and MGT: Designed and conducted the study and drafted the manuscript. All authors have read, reviewed, and approved the final manuscript.

## References

[1] Obeidat B.S, Shdaifat M.M, Aloueedat M.K, Ata M (2022). The effects of feeding chickpea grains on the lactating performance and blood metabolites of ewes. Trop. Anim. Health Prod.

[2] Obeidat B.S (2021). The inclusion of black cumin meal improves carcass characteristics of growing Awassi lambs. Vet. World.

[3] Obeidat B.S (2023). Effect of feeding pomegranate seed pulp on Awassi lambs'nutrient digestibility, growth performance, and carcass quality. Vet. World.

[4] Ismail N, Obeidat B.S (2023). Olive leaves as alternative feed for finishing lambs:Evaluation of feed intake, nutrients digestibility, growth performance, and carcass quality. Italian J. Anim. Sci.

[5] Obeidat B.S, Thomas G.M (2023). Assessing the influence of feeding olive leaves on the productivity and economic viability of growing Awassi lambs. Cogent Food Agric.

[6] Obeidat B.S, Thomas G.M (2024). Growth performance, blood metabolites and carcass characteristics of Black goat kids fed diets containing olive cake. Animals.

[7] Prakash C.V.S, Prakash I (2011). Bioactive chemical constituents from pomegranate (*Punica granatum*) juice, seed and peel-a review. Int. J. Res. Chem. Environ.

[8] Elfalleh W, Hannachi H, Tlili N, Yahia Y, Nasri N, Ferchichi A (2012). Total phenolic contents and antioxidant activities of pomegranate peel, seed, leaf and flower. J. Med. Plants Res.

[9] Lansky E.P, Newman R.A (2007). *Punica granatum* (pomegranate) and its potential for prevention and treatment of inflammation and cancer. J. Ethnopharmacol.

[10] Hamed H.S, Abdel-Tawwab M (2021). Dietary pomegranate (*Punica granatum*) peel mitigated the adverse effects of silver nanoparticles on the performance, haemato-biochemical, antioxidant, and immune responses of Nile tilapia fingerlings. Aquaculture.

[11] Kotsampasi B, Christodoulou C, Mavrommatis A, Mitsiopoulou C, Bampidis V.A, Christodoulou V, Tsiplakou E (2021). Effects of dietary pomegranate seed cake supplementation on performance, carcass characteristics and meat quality of growing lambs. Anim. Feed. Sci. Technol.

[12] Pipatpaitoon N, Paraksa N (2021). Defatted coconut residue as alternative feedstuff for growing and finishing pigs. Songklanakarin. J. Sci. Technol.

[13] Hammond A.C, Wildeus S (1993). Effects of coconut meal or fish meal supplementation on performance, carcass characteristics and diet digestibility in growing St. Croix lambs fed a tropical grass-based diet. Small Rumin. Res.

[14] Food and Agriculture Organization (2021). Socio-economic Analysis and Policy Implications of the Roles of Agriculture in Developing Countries. Summary Report.

[15] Moorthy M, Viswanathan K (2009). Nutritive value of extracted coconut (*Cocos nucifera*) meal. Res. J. Agric. Biol. Sci.

[16] Rokhayati A.U (2020). Coconut meal use as a source of protein vegetable for livestock goat. Artech. J. Innov. Res. Anim. Vet. Sci.

[17] Creswell D.C, Brooks C.C (1971). Composition, apparent digestibility and energy evaluation of coconut oil and coconut meal. J. Anim. Sci.

[18] NRC (2007). Sheep, Goats, Cervids, and New World Camelids.

[19] Obeidat B.S, Al-Khaza'leh J, Alqudah A.M (2023). Black cumin meal (*Nigella sativa*) as an alternative feed resource during the suckling period of Awassi ewes:Assessments of performance and health. Anim. Feed Sci. Technol.

[20] AOAC (1990). Official Methods of Analysis.

[21] El-Elaime R.R (2021). Impact of using different levels of dried pomegranate by-products as feed additives on performance of growing Barki lambs. Egypt. J. Nutr. Feeds.

[22] Sadq S.M, Ramzi D.O.M, Hamasalim H.J, Ahmed K.A (2016). Growth performance and digestibility in Karadi lambs receiving different levels of pomegranate peels. Open J. Anim. Sci.

[23] Shaani Y, Eliyahu D, Mizrahi I, Yosef E, Ben-Meir Y, Nikbachat M, Miron J (2016). Effect of feeding ensiled mixture of pomegranate pulp and drier feeds on digestibility and milk performance in dairy cows. J. Dairy Res.

[24] Modaresi J, Nasri M.H, Rashidi L, Dayani O, Kebreab E (2011). Effects of supplementation with pomegranate seed pulp on concentrations of conjugated linoleic acid and punicic acid in goat milk. J. Dairy Sci.

[25] Shabtay A, Nikbachat M, Zenou A, Yosef E, Arkin O, Sneer O, Miron J (2012). Effects of adding a concentrated pomegranate extract to the ration of lactating cows on performance and udder health parameters. Anim. Feed Sci. Technol.

[26] Hassan F.A, Ibrahim M.R.M, Arafa S.A (2020). Effect of dietary pomegranate by-product extract supplementation on growth performance, digestibility, and antioxidant status of growing rabbit. Trop. Anim. Health Prod.

[27] Jang I.S, Ko Y.H, Kang S.Y, Moon Y.S, Sohn S.H (2007). Effect of dietary supplementation of ground grape seed on growth performance and antioxidant status in the intestine and liver in broiler chickens. Korean J. Poult. Sci.

[28] Faciola A.P, Broderick G.A (2014). Effects of feeding lauric acid or coconut oil on ruminal protozoa numbers, fermentation pattern, digestion, omasal nutrient flow, and milk production in dairy cows. J. Dairy Sci.

[29] Lee C, Hristov A.N, Heyler K.S, Cassidy T.W, Long M, Corl B.A, Karnati S.K.R (2011). Effects of dietary protein concentration and coconut oil supplementation on nitrogen utilization and production in dairy cows. J. Dairy Sci.

[30] Reveneau C, Karnati S.K.R, Oelker E.R, Firkins J.L (2012). Interaction of unsaturated fat or coconut oil with monensin in lactating dairy cows fed 12 times daily. I. Protozoal abundance, nutrient digestibility, and microbial flow to the omasum. J. Dairy Sci.

[31] Hu F, Piao M, Yang C, Diao Q, Tu Y (2023). Effects of coconut oil and palm oil on growth, rumen microbiota, and fatty acid profile of suckling calves. Microorganisms.

[32] Kahraman M.E, Sakar S, Yurtseven A, Das H, Yalcin M, Avci G, Gungoren B, Dogan Das A, Şahan K, Takim B, Erol A.K (2022). The effect of pistachio shell, pomegranate hull, and olive pulp feeding on milk yield, milk quality, and some biochemical blood parameters in sheep. Harran Üniv. Vet. Fak. Derg.

[33] Kazemi M.R, Valizadeh R (2021). The effect of dietary supplementation of ensiled pomegranate by-products on growth performance, nutrient digestibility, hematology parameters and meat characteristics of fat-tail lambs. Italian J. Anim. Sci.

[34] Muhlisin M, Noviandi C.T, Utomo R, Yusiati L.M (2016). The Effects of Coconut Meat Waste as Feed Alternative in Sheep Ration on Cholesterol Content and Meat Quality. Conference. The 17^th^ Asian-Australasian Association of Animal Production Societies Animal Science Congress.

[35] Durand D, Bauchart D, Picherit C, Gruffat D, Graulet B (1998). Effects of dietary coconut oil on the density distribution and the chemical composition of plasma lipoproteins in the preruminant calf. Reprod. Nutr. Dev.

[36] Zeweil H.S, Ahmed M.H, Zahran S.M, El-Gindy Y.M, Abdulgader E.A.S (2016). Effect of pomegranate peel addition to the diet enriched with linseed oil on performance, lipid traits in the meat, blood lipid profile and antioxidant property of rabbits under summer conditions. Egypt. J. Nutr. Feeds.

[37] Khan K.M.H, Hamasalim H.J, Sadq S.M, Ramzi D.O.M (2015). Changes in lipid profile and some blood biochemical parameters in Karadi lambs receiving different levels of pomegranate peels. Res. Opin. Anim. Vet. Sci.

[38] Aviram M, Rosenblat M (2013). Pomegranate for your cardiovascular health. Rambam Maimonides Med. J.

[39] Aregheore E.M (2006). Utilization of concentrate supplements containing varying levels of copra cake (*Cocos nucifera*) by growing goats fed a basal diet of Napier grass (*Pennisetum purpureum*). Small Rumin. Res.

[40] Hennessy D.W, Kempton T.J, Williamson P.J (1989). Copra meal as a supplement to cattle offered a low quality native pasture hay. Asian-Aust. J. Anim. Sci.

[41] Emami A, Nasri M.F, Ganjkhanlou M, Rashidi L, Zali A (2015). Dietary pomegranate seed pulp increases conjugated-linoleic and-linolenic acids in muscle and adipose tissues of kid. Anim. Feed Sci. Technol.

[42] Kotsampasi B, Christodoulou V, Zotos A, Liakopoulou-Kyriakides M, Goulas P, Petrotos K, Bampidis V.A (2014). Effects of dietary pomegranate by-product silage supplementation on performance, carcass characteristics and meat quality of growing lambs. Anim. Feed Sci. Technol.

[43] Galgal K.K, McMeniman N.P, Norton B.W (1994). Effect of copra expeller pellet supplementation on the flow of nutrients from the rumen of sheep fed low-quality Pangola grass (*Digitaria decumbens*). Small Rumin. Res.

